# Substitution of self-reported measures for objectively assessed grip strength and slow walk in the Physical Frailty Phenotype: ramifications for validity

**DOI:** 10.1186/s12877-023-04105-8

**Published:** 2023-07-22

**Authors:** Karen Bandeen-Roche, Jing Tian, Brian Buta, Jeremy Walston, Qian-Li Xue

**Affiliations:** 1 Bloomberg School of Public Health, Johns Hopkins University, Baltimore, MD USA; 2 School of Medicine, Johns Hopkins University, Baltimore, MD USA; 3 Johns Hopkins Center On Aging and Health, Johns Hopkins University, Baltimore, MD USA; 4 School of Nursing, Johns Hopkins University, Baltimore, MD USA

**Keywords:** Aging, Construct validation, Diagnostic accuracy, Measurement error, Physical function, Vulnerability

## Abstract

**Background:**

Frailty assessment promises to identify older adults at risk for adverse consequences following stressors and target interventions to improve health outcomes. The Physical Frailty Phenotype (PFP) is a widely-studied, well validated assessment but incorporates performance-based slow walk and grip strength criteria that challenge its use in some clinical settings. Variants replacing performance-based elements with self-reported proxies have been proposed. Our study evaluated whether commonly available disability self-reports could be substituted for the performance-based criteria in the PFP while still identifying as “frail” the same subpopulations of individuals.

**Methods:**

Parallel analyses were conducted in 3393 female and 2495 male Cardiovascular Health Study, Round 2 participants assessed in 1989–90. Candidate self-reported proxies for the phenotype’s “slowness” and “weakness” criteria were evaluated for comparable prevalence and agreement by mode of measurement. For best-performing candidates: Frailty status (3 + positive criteria out of 5) was compared for prevalence and agreement between the PFP and mostly self-reported versions. Personal characteristics were compared between those adjudicated as frail by (a) only a self-reported version; (b) only the PFP; (c) both, using bivariable analyses and multinomial logistic regression.

**Results:**

Self-reported difficulty walking ½ mile was selected as a proxy for the phenotype’s slowness criterion. Two self-reported weakness proxies were examined: difficulty transferring from a bed or chair or gripping with hands, and difficulty as just defined or in lifting a 10-pound bag. Prevalences matched to within 4% between self-reported and performance-based criteria in the whole sample, but in all cases the self-reported prevalence for women exceeded that for men by 11% or more. Cross-modal agreement was moderate, with by-criterion and frailty-wide Kappa statistics of 0.55–0.60 in all cases. Frail subgroups (a), (b), (c) were independently discriminated (*p* < 0.05) by race, BMI, and depression in women; by age in men; and by self-reported health for both.

**Conclusions:**

Commonly used self-reported disability items cannot be assumed to stand in for performance-based criteria in the PFP. We found subpopulations identified as frail by resultant phenotypes versus the original phenotype to systematically differ. Work to develop self-reported proxies that more closely replicate their objective phenotypic counterparts than standard disability self-reports is needed.

**Supplementary Information:**

The online version contains supplementary material available at 10.1186/s12877-023-04105-8.

## Background

The burgeoning of our older adult population augurs escalating burden of disease, disability, and adverse events with consequential personal and societal costs [1]. Older adults are not a monolithic at-risk population, however, but have highly heterogeneous health status and prospects [2, 3]. Methods to distinguish those most likely to need support services or experience health crises are urgently needed if the impending burden is to be addressed effectively and efficiently. Frailty—a clinical state of increased vulnerability to adverse health outcomes when faced with stressors [4], characterized in a 2013 Lancet article as “the most problematic expression of population ageing” [5]—fits the bill for characterizing the at-risk individuals and, potentially, for targeting interventions to improve health outcomes. Frailty assessment to guide patient management in the context of major medical procedures has begun to be implemented in selected sub-specialties (e.g. [6–9]). A consensus panel has recommended that all individuals older than 70 years of age should be screened for frailty [4].

Among a large diversity of methods available to practitioners for assessing frailty, we focus here on the Physical Frailty Phenotype (PFP) proposed by Fried and colleagues in 2001 [10]. It appeals to us because it is not only designed to identify persons at risk but also to reflect specific physiologic etiology that might itself be addressed, so as to delay frailty onset in the first place [11]. It comprises 5 “criteria” assessing exhaustion, low physical activity, weight loss, slow walk, and weak grip strength: In its standard operationalization, the last two are objectively measured by performance testing referenced against standard office visit measures (height and—to obtain body mass index—weight), and the others are measured by self-report [10]. Assessment takes roughly 15–20 min [12].

Whereas PFP assessment is relatively convenient and is the method whose use has been most frequently reported in publications [13], some voice concerns over feasibility in clinical practice. Fifteen minutes may not be expendable in a busy clinical setting. Objective testing is infeasible in certain situations, such as an emergent health crisis, and it does not permit retrospective ascertainment. Consequently, a number of instruments purporting to simplify the PFP have been proposed. Theou and colleagues surveyed this landscape (as well as other modifications) in a 2015 article [14]: Among original research articles identified in a systematic review as using the PFP to assess frailty, roughly 10% used standard self-reported disability items querying difficulty with upper-extremity, ADL, or mobility tasks to replace performance-based assessment of strength or slow walk (each). In this same publication, Theou and colleagues conducted a de novo analysis replacing the phenotype’s original strength and slow walk criteria with self-reported disability items (among many other variations) in the Survey of Health, Ageing, & Retirement in Europe: agreement was found to be mediocre (Kappa = 0.44). A well-known instrument of relevance not included in this publication’s literature review is the Tilburg Frailty Indicator, which includes a physical components subscale to assess physical aspects of frailty, including the PFP constructs. In an article evaluating its psychometric properties, its questions on mobility and strength achieved moderate Pearson’s correlations of 0.35–0.36 with performance-based alternative measures [15].

In studies replacing performance-based PFP criteria with self-reported counterparts, replacement has been justified by ability of the self-reported PFP to predict adverse outcomes [16] or by reasonable association with a standard PFP measurement [17]. If screening for high risk is one’s only goal, such justifications may suffice. However, if one’s interest ultimately is to intervene so as to delay or prevent frailty itself, and not only to manage persons deemed to be vulnerable, then validity also becomes an important consideration. This is the focus of our paper: specifically, to evaluate whether the PFP as originally proposed and a version substituting its performance-based criteria with standard self-reported disability items identify the same subpopulations of individuals. To assess this, we analyzed data from the Cardiovascular Health Study, using comparisons of personal characteristics across persons deemed frail by either, or both, methods. Like Theou and colleagues, we also compared versions for agreement, but we do not consider this primarily a validity assessment as two methods could exhibit weak agreement and yet identify the same subpopulations, if primarily distinguished by “random” measurement error.

## Methods

### Study sample

In brief, CHS was an epidemiological cohort study designed to identify risk factors for the development and progression of cardiovascular disease in older adults [18, 19]. As reported on the CHS website https://chs-nhlbi.org/CHSOverview: Ultimately 5888 adults aged 65 + participated. Baseline examinations consisted of a home interview and a clinic examination; thereafter there were annual clinic assessments of participants spanning 10 years. Measures collected annually or nearly annually addressed traditional cardiovascular risk factors, subclinical cardiovascular disease, medication use, cognitive and physical function, depression, and personal characteristics and history. Additional characteristics were assessed at less frequent intervals. Measures needed to construct the PFP were available at visits 2 (1989–1990), 5 (1992–1993), and 9 (1996–1997).

Our analysis utilized data from visit 2. We performed it stratified by sex (*n* = 3393 females and *n* = 2495 males).

### Measures

#### Frailty

The PFP paradigm is based on five criteria: exhaustion, low physical activity, weakness, slowness, and shrinking. The foundational study developing the PFP used CHS data: Criteria we analyzed were operationalized as described in that study (Table 1), with slight modifications to weakness, slowness and shrinking that we motivate and describe shortly. In the foundational study, criteria for exhaustion and low physical activity were self-reported (detail provided in Table 1); weakness was assessed by handgrip strength in the dominant hand using a Jamar handheld dynamometer (3 measures averaged), and slowness was assessed by usual walking pace measured over a 15-foot course [10]. For our study, persons not able to complete the weakness and slowness assessments for health or physical reasons were scored as having the criterion [20]. In the originally proposed PFP paradigm, shrinking was assessed by self-report of unintentional weight loss (see Table 1). Subsequent PFP implementations have recognized the potential for a floor effect on weight changes by alternatively assigning a “shrinking” criterion for underweight status [21]: we did likewise (Table 1).

**Table 1:** Frailty-defining criteria and prevalence by sex

**Characteristics**	**Women (*n* = 3393)**		**Men (*n* = 2495)**	
	**Definition**	**%**		**%**
1.** Weight loss**	Self-report of “yes” to the question “In the last year, have you lost more than 10 pounds unintentionally (i.e., not due to dieting or exercise)?” OR Body Mass Index ≤ 18.5 kg/m^2^	8.4	As for women	7.8
2.** Exhaustion**	Self-report of “a moderate amount” or “most of the time” (versus “rarely or none of the time” or “some or a little of the time”) to either of: i) “I felt that everything I did was an effort” ii) “I could not get going” Query was specified “How often in the last week did you feel this way?”	21.4	As for women	14.7
3. **Low Energy Expenditure**	< 270 Kcals/week as self-reported using Minnesota Leisure Time Activity scale and estimated using that instrument’s algorithm (18 items)	24.6	< 383 Kcals/week on Minnesota Leisure Time Activity scale and estimated using that instrument’s algorithm	21.4
4.** Slowness**	Walking 15 feet (4.57 m) time > 7 s for height < = 159 cm time > 6 s for height > 159 cm	18.8	Walking 15 feet (4.57 m) time > = 7 for height < = 173 cm time > = 6 for height > 173 cm	26.3
5.** Weakness**	Grip strength < = 16.7 kg for BMI < = 23 kg/m^2^ < = 17.7 kg for BMI 23.1 – 26 kg/m^2^ < = 17.7 kg for BMI 26.1 – 29 kg/m^2^ < = 18.3 kg for BMI > 29 kg/m^2^	23.0	Grip strength < = 28.3 kg for BMI < = 24 kg/m^2^ < = 30.0 kg for BMI 24.1—26 kg/m^2^ < = 30.0 kg for BMI 26.1—28 kg/m^2^ < = 31.3 kg for BMI > 28 kg/m^2^	21.3

As described in the foundational paper [10], slowness and weakness criteria were designed to capture the lowest 20% of measured performance within sex-by-height (for slowness) or by-BMI (for weakness) categories. In both cases, however, our preliminary analyses revealed that well over 20% of CHS participants met the criteria published in the foundational paper at the baseline frailty assessment. Therefore, we adjusted the criteria to accomplish as nearly 20% yield as possible, which had the effect of making them modestly more stringent (Table 1).

We then proceeded to identify self-reported disability items as candidates to replace the weakness and slowness criteria in the PFP. Among items assessed in the CHS: we considered self-reported difficulty with upper extremity tasks as potential replacements for the weakness criterion using (i) the either-or-both combination of difficulty getting out of a bed or chair with difficulty gripping with hands; (ii) difficulty lifting or carrying a 10-pound bag of groceries; and (iii) the either-or-both combination of (i) and (ii). As potential replacements for the slowness criterion, we considered self-reported mobility limitation using: (i) difficulty walking half a mile and (ii) its either-or-both combination with difficulty climbing a flight of stairs.

For both the original PFP (henceforth, PFP) and for versions replacing objectively measured items with self-reported counterparts (henceforth, SPFP), “frail status” was assigned using the number of criteria met: those with none were considered “robust;” those with 1–2, “pre-frail;” and those with 3–5, “frail.”

#### Personal characteristics potentially discriminating frail characterization by PFP and SPFP

Demographic variables considered were self-reported age in years, race (black versus non-black), education (years), and marital status (married, widowed, divorced/separated, never married). Disease burden was considered as number of diseases among rheumatoid arthritis, diabetes, cancer, myocardial infarction (MI), angina, congestive heart failure (CHF). Of the diseases assessed, four including angina, myocardial infarction, congestive heart failure, and peripheral vascular disease were adjudicated by a panel of experts reviewing medical records and scored as definite or non-definite [22]. All other diseases were based on self-report. Other measures of health and cognitive status were self-reported health (excellent, very good, good, fair, poor), BMI (measured in clinic), depression symptomatology as assessed by the modified 10-item Center for Epidemiological Studies-Depression scale (CES-D-10) [23], and Mini-Mental State Examination (MMSE) [24] score.

### Statistical analysis

Descriptive analyses were applied to characterize our sample. Two-sample tests were used to compare personal characteristics by sex, employing t-tests for continuously measured characteristics and chi-square tests for categorical characteristics. Descriptive analyses then were conducted to inform selection of self-reported substitutes for weakness and slowness criteria of the PFP. We sought to avoid differentiating the PFP and SPFPs by virtue of largely varying prevalence between original and substitute items, and so emphasized similarity of prevalence in selecting self-reported criterion substitutions. To ensure that this strategy did not miss substitute criteria achieving clearly superior agreement with original criteria or result in clearly inferior diagnostic inaccuracy for approximating the PFP, we evaluated item characteristic curves [25] describing prevalence of each self-reported criterion by grip strength and walking speed, obtained by smoothing scatterplots of each SPFP criterion versus its grip strength or walking speed counterpart using the smoothing splines method [26], and receiver operating characteristic (ROC) curves assessing the accuracy of predicting each candidate SPFP candidate criteria by its strength or walking speed counterpart.

Concordance of the original PFP with the resulting self-reported PFP was evaluated using cross-tabulations and weighted kappa statistics with Cicchetti-Allison weights (a.k.a. Linear weights). We also conducted sensitivity analyses motivated by consideration of the decades that have passed since the CHS data were collected: Cohen’s kappa is known to be sensitive to both prevalence of the condition under assessment (i.e. frailty) and differences in classification probabilities (i.e., assessment bias) between raters (or instruments, i.e., self-report vs. performance). It is conceivable that the prevalence of frailty has changed over time with population aging, and evolving self-report behavior independent of frailty status is an example of assessment bias. Given that more extremely high or low prevalence of frailty and greater assessment bias could deflate and inflate kappa respectively for CHS versus a present day counterpart despite the same percentage agreement [27], we calculated sex-specific prevalence and bias-adjusted kappa (PABAK) [27, 28] in our study sample. PABAK adjusts for between-method bias by replacing the two discordant probabilities by their average and for between-method prevalence differences by also replacing the two concordant probabilities by their average, which effectively constrains the chance agreement probability to 0.5 such that PABAK becomes a function of the probability of observed agreement only. We also calculated PABAK in a hypothetical sample with the same sex-specific sample size as in the CHS but a higher prevalence of 15.3% being frail and 45.5% being prefrail overall; 17.2% being frail and 47.2% being prefrail (by PFP) among females and 12.9% being frail and 43.3% being prefrail among males as seen in a nationally representative sample of U.S. Medicare beneficiaries aged 65 years and older [29].

To adjudicate whether the PFP and selected SPFP identify similar (potentially imprecisely) or distinct subpopulations as “frail,” we conducted descriptive and regression analyses to identify characteristics distinguishing three “frail” subgroups: ones identified as frail by (i) the original PFP but not the SPFP; (ii) the SPFP but not the original PFP; and (iii) both PFP and SPFP. Demographic characteristics and health indicators were compared across the three groups, using ANOVA and chi-squared tests respectively for continuously and categorically measured discriminating variables. Multinomial logistic regression was performed to explore independent associations.

All analyses were conducted using SAS version 9.4.

## Results

Table 2 characterizes demographic and health information in our analytic cohort. Distributions of nearly all characteristics differed strongly between men and women, with men 0.8 year older and 0.7 year better educated on average and having nearly an additional 29% married and 9% with income > $35,000. Men also tended to have more diseases (0.2 higher mean number of diseases and cardiovascular disease and diabetes prevalence higher by 59% and 26% respectively). Women reported higher prevalence of depression and rheumatoid arthritis. BMI, self-reported health, and cancer prevalence were similar between sexes. Overall the cohort tended toward the younger-old (mean age 72.8 years), being highly educated (mean 13.7 years), and white (84.1%).

**Table 2:** Sample characteristics by sex

	Overall *N* = 5888	Men *N* = 2495	Female *N* = 3393	*P*-value^*^
Age (years), mean (std)	72.8 (5.6)	73.3 (5.7)	72.5 (5.5)	< 0.001
Education (years), mean (std)	13.7 (4.8)	14.1 (5.1)	13.4 (4.5)	< 0.001
MMSE, mean (std)	89.6 (7.3)	89.2 (7.5)	89.9 (7.1)	< 0.001
# of diseases, mean (std)^a^	1.4 (1.3)	1.5 (1.4)	1.3 (1.2)	< 0.001
BMI, mean (std)	26.7 (4.7)	26.4 (3.8)	26.9 (5.3)	< 0.001
Race(black), n (%)	933 (15.9)	347 (13.9)	586 (17.3)	< 0.001
Marriage, n (%)				< 0.001
Married	3893 (66.2)	2064 (82.8)	1829 (54.0)	
Widowed	1449 (24.6)	261 (10.5)	1188 (35.0)	
Separated/divorced/never married	540 (9.2)	167 (6.7)	373 (11.0)	
# of depressive symptoms, mean (std)	4.7 (4.6)	3.9 (4.2)	5.3 (4.8)	< 0.001
Income				< 0.010
< $16,000	2324 (42.2)	762 (32.0)	1562 (49.9)	
$16,000–35,000	1925 (34.9)	948 (39.9)	977 (31.2)	
> $35,000	1259 (22.9)	668 (28.1)	591 (18.9)	
Health status, n (%)				0.038
excellent	790 (13.5)	362 (14.5)	428 (12.6)	
very good	1415 (24.1)	592 (23.8)	823 (24.3)	
good	2175 (37.0)	944 (37.9)	1231 (36.4)	
fair	1256 (21.4)	500 (20.1)	756 (22.3)	
poor	239 (4.1)	91 (3.7)	148 (4.4)	
Cancer, n (%)	840 (14.3)	362 (14.5)	478 (14.1)	0.650
Congestive heart failure, n (%)	275 (4.7)	133 (5.3)	142 (4.2)	0.040
Myocardial infarction, n (%)	562 (9.5)	350 (14.0)	212 (6.3)	< 0.001
Coronary heart disease, n (%)	1154 (19.6)	622 (24.9)	532 (15.7)	< 0.001
Rheumatoid arthritis, n (%)	3025 (52.0)	1090 (44.2)	1935 (57.7)	< 0.001
Diabetics, n (%)	1740 (29.9)	838 (33.9)	902 (26.9)	< 0.001
Angina, n (%)	964 (16.4)	498 (20.0)	466 (13.7)	< 0.001

^a^Diseases included are: rheumatoid arthritis, diabetes, cancer, congestive heart failure, myocardial infarction, coronary heart disease, angina

^*^For continuous variables, t-tests were used for inferences; for categorical variables, Chi-square tests were used

Table 3 compares proportions judged to be “slow” and “weak” by performance-based criteria, overall and by gender. The proposed performance-based criteria successfully identified roughly the bottom quintile of gait speed and grip strength, with 22.0% judged as “slow” and 22.3% judged as “weak.” Percentages adjudicated “slow” were considerably higher for men (26.3%) than women (18.8%), while percentages adjudicated “weak” were more similar between sexes (21.3% for men, 23.1% for women).

**Table 3:** Criterion prevalence for performance-based criteria and self-reported substitution candidates, by sex

Criterion	Measure	Number with positive criterion (% of total)		
		Overall	Women	Men
Slowness	Performance-based	1279 (22.0)	630 (18.8)	649 (26.3)
	Difficulty walking ½ mile	1230 (21.2)	841 (25.2)	389 (15.8)
	Difficulty walking ½ mile or climbing 10 steps	1431 (24.4)	987 (29.2)	444 (17.9)
Weakness	Performance-based	1266 (22.3)	755 (23.1)	511 (21.3)
	Difficulty lifting a 10-pound bag	853 (14.6)	723 (21.6)	130 (5.3)
	Difficulty gripping with hands	896 (15.3)	663 (19.7)	233 (9.4)
	Difficulty gripping or transferring	1079 (18.4)	794 (23.5)	285 (11.5)
	Difficulty gripping, transferring or lifting a 10-pound bag	1547 (26.4)	1186 (35.0)	361 (14.5)

Table 3 also reports prevalence of the various self-reported substitution items we considered. Among substitutes for slowness, the percentage reporting difficulty in walking ½ mile (21.2%) most closely matched the percentage meeting the frailty criterion for its performance-based counterpart. For weakness, two items achieved a comparably-closest match in percentage with the criterion: transferring from a bed or chair, gripping with hands, or lifting and carrying a 10-pound bag (26.4%; henceforth, weakness version “TGL”), and difficulty transferring from a bed or chair or gripping with hands (18.4%; henceforth, version “TG”). We carried forward both versions for comparison. For each criterion and version just noted, men’s self-reports were substantially less frequently “frail” than women’s, despite that percentages judged as frail by performance-based criteria were comparable or even higher for men than women. For women, self-reported weakness was considerably more prevalent by the TGL criterion (35.0%) than the performance-based criterion (23.1%) whereas for TG there was a close match (23.5% self-reported); the relative closeness for men was reversed (14.5% with self-reported weakness for TGL; 11.5% for TG versus 21.3% performance-based).

**Fig. 1:**
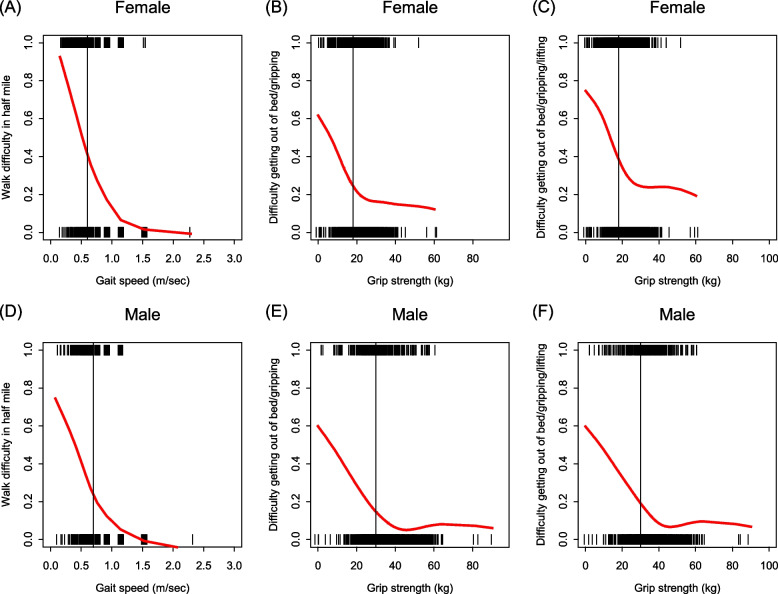
Self-reported slowness and weakness criteria prevalence by objective counterpart measure values. Plots show the probability of being positive on the self-reported criterion as a function of the performance-based counterpart used to assess slowness or weakness in the original PFP, estimated using a smoothing spline with 5 degrees of freedom. Panels going left to right respectively are **A** and **D** for slowness (difficulty walking ½ mile on y-axis, measured gait speed on x-axis), **B** and **E** weakness (difficulty transferring or gripping on y-axis, measured grip strength on x-axis), and **C** and **F** our alternative weakness comparison (difficulty transferring, gripping, or lifting on y-axis, measured grip strength on x-axis). Females are shown at the top and males at the bottom. Vertical lines show the performance-based cutoff defining having the criterion (values less than line on the x-axis)

Figure 1 estimates—by gender—the proportion meeting self-reported slowness and weakness criteria as a function, respectively, of gait speed and grip strength. Each plot is annotated with a vertical black line (reference) marking approximately the cutoff for meeting frailty criteria for each performance-based measure: For a nearly ideally performing substitute, such an “item characteristic curve” should be inverse S-shaped, with self-reported prevalence predominantly near 1 below the black line, decreasing steeply in the neighborhood of the black line, and predominantly near 0 above the black line [25] As desired: For each of the figures, the performance-based reference did indeed fall in a neighborhood of most steeply decreasing self-reported criterion prevalence. Less ideally: None of the plots showed self-reported criterion prevalence predominantly near 1 for values of worse performance than the reference, but rather following a reasonably linear trend with worsening performance. For all but slowness in females, the maximum self-reported prevalence observed reached only 0.5–0.7, indicating considerable failure to report difficulty even at the worst possible performance. In ROC analysis, the estimated area under the curve for predicting each self-reported criterion by its respective performance based measures was 0.72 for slowness (overall; similar for women and men) 0.67 for the TG weakness measure (similar in men but considerably worse in women, at 0.60), and 0.69 for the TGL weakness measure (0.67 in men and 0.61 in women). Kappa values were 0.25 for objective versus self-reported slowness criteria overall (95% confidence interval—CI—0.22 to 0.28), 0.21 for men (95% CI 0.17 to 0.26) and 0.29 for women (95% CI 0.25 to 0.33). Kappa values were 0.56 to 0.58 overall and for both sexes for the TG weakness item and 0.56 for all three groups for the TGL weakness (with 95% CI width between 0.03 and 0.06 in all cases). These values indicate that self-reported slowness and weakness criteria are no better than moderately well discriminated by the performance measures underlying their objective counterparts.

Table 4 cross tabulates frailty as assessed by the PFP versus the SPFP with version TG for weakness (non-frail, pre-frail, frail). No differential classification of persons frail by one method as non-frail by another method was observed. Differential classification across adjacent categories, however, was common—particularly in which frailty was less severe when assessed by SPFP than by PFP: 21.9% of women and 35.3% of men judged prefrail by PFP were judged non-frail by SPFP, and 39.2% of women and 57.1% of men judged frail by PFP were judged prefrail by SPFP. In males, misclassification in the reverse direction was far less frequent. Kappa values were 0.56 in the overall sample and ranged from 0.55 in men to 0.57 in women, consistent with other published studies [14]. There was a notable improvement in Kappa after adjusting for prevalence and assessment bias, with PABAK = 0.67 in women, and 0.68 in men and the overall sample (Table 4). Analyses using version TGL for weakness yielded similar Kappa values and patterns of misclassification in men, but much more balanced misclassification across adjacent categories for women (Supplemental Table S1). In the sensitivity analysis using the hypothetical sample, increasing prevalence of frailty had little impact on the crude and adjusted Kappas (Supplemental Table S2).

**Table 4:** Agreement between physical frailty phenotypes: original assessment versus with self-reported substitutions

	Frailty defined by objective measures	Frailty defined by self-report^a^ measures			Kappa (95% CI)
		Nonfrail (*n* = 2905)	Prefrail (*n* = 2476)	Frail (*n* = 503)	
Overall	Nonfrail (*n* = 2516)	2102 (83.6)^b^, (72.4)^c^	414 (16.5), (16.7)	0 (0.0), (0.0)	Kappa with Linear Weighting 0.56 (0.54–0.58) PABAK^d^ 0.68 (0.66–0.69)
(*N* = 5884)	Prefrail (*n* = 2870)	795 (27.7), (27.4)	1834 (63.9), (74.1)	241 (8.4), (47.9)	
	Frail (*n* = 498)	8 (1.6), (0.3)	228 (45.8), (9.2)	262 (52.6), (52.1)	
Female		Nonfrail (*n* = 1493)	Prefrail (*n* = 1534)	Frail (*n* = 363)	
(*N* = 3390)	Nonfrail (*n* = 1443)	1130 (78.3), (75.7)	313 (21.7), (20.4)	0 (0.0), (0.0)	Kappa with Linear Weighting 0.57 (0.55–0.59) PABAK 0.67 (0.66–0.69)
	Prefrail (*n* = 1631)	358 (21.9), (24.0)	1097 (67.3), (71.5)	176 (10.8), (48.5)	
	Frail (*n* = 316)	5 (1.6), (0.3)	124 (39.2), (8.1)	187 (59.2), (51.5)	
Male		Nonfrail (*n* = 1412)	Prefrail (*n* = 942)	Frail (*n* = 140)	
(*N* = 2494)	Nonfrail (*n* = 1073)	972 (90.6), (68.8)	101 (9.4), (10.7)	0 (0.0), (0.0)	Kappa with Linear Weighting 0.55 (0.52–0.57) PABAK 0.68 (0.66–0.70)
	Prefrail (*n* = 1239)	437 (35.3), (31.0)	737 (59.5), (78.2)	65 (5.3), (46.4)	
	Frail (*n* = 182)	3 (1.7), (0.2)	104 (57.1), (11.0)	75 (41.2), (53.6)	

^a^Weakness assessed by report of difficulty gripping with hands or transferring from a bed or chair; Slowness assessed by report of difficulty of walking one-half a mile

^b^Row percentage

^c^Column percentage

^d^PABAK: Prevalence and Bias Adjusted Kappa that assigns partial credit of 1/2 to misses by 1 category

Tables 5 and 6 compare distributions of demographic and disease characteristics across persons deemed frail by either method singly or by both together (3 groups) for the self-reported frailty version yielding the more conservative findings—the one incorporating TGL weakness assessment. For women, determinants most strongly distinguishing the groups (*p* < 0.001) were *age* (those deemed frail by the standard PFP nearly 3 years older on average than those deemed frail only by the self-report version), *BMI* (those deemed frail by the self-report version only heavier than others by 2.2–3.4 kg/m^2^), *depression symptoms* (those deemed frail by the self-report version 2 symptoms higher than those frail by the original PFP only), and *self-reported health* status (percentage reporting poor self-reported health ranged stepwise from 5 to 26% for those frail by the original PFP only, self-report only, and both assessments) (Table 5). Self-reported health distinguished men across frailty groups similarly as for women; age and depression associations were in the same direction, but somewhat less strongly evidenced (*p*-values 0.009, 0.004 respectively) (Table 6).

**Table 5:** Characteristics comparison across females judged frail by either method only or by both^a^

	Frail by self-report PFP only (*N* = 244)	Frail by objective PFP only (*N* = 98)	Frail by both (*N* = 218)	*P*-value*
Age (years), mean (std)	73.9 (6.4)	76.7 (7.0)	76.3 (6.0)	< 0.001
Education (years), mean (std)	12.2 (4.4)	12.0 (5.1)	12.2 (4.9)	0.950
MMSE, mean (std)	88.5 (7.0)	81.9 (13.0)	85.5 (9.3)	< 0.001
# of disease*, mean (std)	2.0 (1.4)	1.5 (1.3)	2.1 (1.4)	0.004
Body Mass Index, mean (std)	29.5 (6.5)	26.1 (5.6)	27.3 (7.1)	< 0.001
Race(black), n (%)	46 (18.9)	43 (43.9)	56 (25.7)	< 0.001
Marriage, n (%)				0.013
Married	125 (51.2)	35 (35.7)	83 (38.1)	
Widowed	94 (38.5)	47 (48.0)	97 (44.5)	
Separated/divorced/never married	25 (10.3)	16 (16.3)	38 (17.4)	
# of depressive symptoms, mean (std)	8.8 (5.6)	6.8 (5.3)	10.3 (6.0)	< 0.001
Income				0.038
< $16,000	137 (60.1)	68 (74.7)	134 (68.0)	
$16,000–35,000	66 (29.0)	12 (13.2)	41 (20.8)	
> $35,000	25 (11.0)	11 (12.1)	22 (11.2)	
Health status, n (%)				< 0.001
excellent	7 (2.9)	3 (3.1)	6 (2.8)	
very good	23 (9.4)	15 (15.5)	18 (8.3)	
good	62 (25.8)	33 (34.0)	44 (20.2)	
fair	113 (46.3)	41 (42.3)	93 (42.7)	
poor	38 (15.6)	5 (5.2)	57 (26.2)	
Cancer, n (%)	48 (19.7)	10 (10.2)	38 (17.4)	0.109
Congestive heart failure, n (%)	23 (9.4)	9 (9.2)	31 (14.2)	0.206
Myocardial infarction, n (%)	26 (10.7)	8 (8.2)	21 (9.6)	0.777
Coronary heart disease, n (%)	69 (28.3)	19 (19.4)	65 (29.8)	0.142
Rheumatoid arthritis, n (%)	188 (78.0)	62 (63.9)	172 (81.5)	0.008
Diabetics, n (%)	88 (36.4)	32 (33.3)	77 (36.7)	0.835
Angina, n (%)	64 (26.2)	16 (16.3)	57 (26.2)	0.119

^a^Weakness self-reported substitution = transferring, gripping and lifting

^*^For continuous variables, ANOVA was used to calculate and for categorical variables, Chi-square test was used

**Table 6:** Characteristics comparison across males judged frail by either method only or by both^a^

	Frail by self-report only (*N* = 82)	Frail by objective only (*N* = 98)	Frail by both (*N* = 84)	*P*-value*
Age (years), mean (std)	74.7 (6.5)	77.6 (6.5)	77.0 (6.7)	0.009
Education (years), mean (std)	12.0 (5.1)	12.9 (5.0)	12.3 (5.2)	0.524
MMSE, mean (std)	86.2 (7.3)	85.5 (9.6)	83.7 (11.9)	0.262
# of disease*, mean (std)	2.2 (1.5)	1.9 (1.4)	2.4 (1.7)	0.156
Body Mass Index, mean (std)	26.3 (4.1)	26.0 (3.9)	26.3 (4.5)	0.849
Race(black), n (%)	16 (19.5)	25 (25.5)	16 (19.1)	0.492
Marriage, n (%)				0.480
Married	64 (78.1)	74 (75.5)	56 (66.7)	
Widowed	13 (15.9)	15 (15.3)	19 (22.6)	
Separated/divorced/never married	5 (6.1)	9 (9.2)	9 (10.7)	
# of depressive symptoms, mean (std)	7.3 (5.3)	6.4 (4.5)	9.0 (6.1)	0.004
Income				0.740
< $16,000	39 (50.6)	44 (47.3)	35 (43.8)	
$16,000–35,000	26 (33.8)	29 (31.2)	31 (38.7)	
> $35,000	12 (15.6)	20 (21.5)	14 (17.5)	
Health status, n (%)				< 0.001
excellent	2 (2.5)	3 (3.1)	0 (0)	
very good	4 (5.0)	11 (11.2)	9 (10.7)	
good	28 (35.0)	46 (46.9)	21 (25.0)	
fair	26 (32.5)	36 (36.7)	29 (34.5)	
poor	20 (25.0)	2 (2.0)	25 (29.8)	
Cancer, n (%)	15 (18.3)	17 (17.4)	10 (12.1)	0.471
Congestive heart failure, n (%)	14 (17.1)	8 (8.2)	18 (21.4)	0.038
Myocardial infarction, n (%)	17 (20.7)	16 (16.3)	21 (25.0)	0.351
Coronary heart disease, n (%)	27 (32.9)	25 (25.5)	34 (40.5)	0.099
Rheumatoid arthritis, n (%)	56 (68.3)	54 (55.7)	58 (70.7)	0.086
Diabetics, n (%)	29 (35.4)	48 (49.5)	31 (38.3)	0.120
Angina, n (%)	22 (26.8)	21 (21.4)	29 (34.5)	0.141

^a^Weakness self-reported substitution = transferring, gripping and lifting

^*^For continuous variables, ANOVA was used to calculate and for categorical variables, Chi-square test was used

We also conducted multivariable multinomial logistic regression analyses of frailty type (3 groups) simultaneously on the characteristics in Table 2. All the associations named in the previous paragraph as most strongly distinguishing except age for women and depression for men remained independently statistically significant (*p* < 0.05), whether including all characteristics shown in Table 2 in models, or applying forward (entry = 0.05), backward (removal = 0.10), or full (entry = removal = 0.10) stepwise selection to identify, and remove, collinear or non-predictive variables. Race emerged as an additional independent discriminant for women (odds of being found frail only by the original PFP vs. by both methods increased 2.63-fold in black versus white older adults, with 95% confidence interval 1.31- to 5.28-fold, after controlling for age, BMI, depression score and self-report of health).

Findings were similar when considering the self-reported frailty version with the TG rather than the TGL self-reported weakness assessment (Supplemental Tables S1, S3, S4). When considering potentially discriminating characteristics one at a time, # of diseases discriminated the groups modestly more strongly for both men and women. Tellingly, also for both women and men, rheumatoid arthritis considerably more strongly discriminated groups when employing the TG weakness assessment, exhibiting prevalence 18 points higher in those frail by the self-reported assessment than the original PFP only. Multivariable findings varied only slightly from those reported in the previous paragraph: RA emerged as an additional discriminant of frailty groups for women.

## Discussion

In a large epidemiologic cohort of older adults, we found novel evidence that populations identified as frail systematically differ when identified by the Physical Frailty Phenotype (PFP) versus a version replacing performance-based measures of slowness and weakness by self-reported disability counterparts. Among women, better self-reported health, self-identifying as Black, lower cognitive performance and fewer depressive symptoms were independently positively associated with being found frail by the original PFP but not by the SPFP we examined. Higher BMI and younger age were positively associated being found frail by the SPFP we examined but not the original PFP, with the BMI association persisting independently. Similar associations with self-reported health and age were evidenced for men—for them, both of these associations persisted independently. As a second novel finding, our data evidenced that self–report assessment which well- or over-approximates the objectively-assessed percentage meeting frailty criteria in women substantially *under*estimates the same in men. Finally, our study reiterated extant findings that PFP assessment using the original criteria for slow walk and weakness versus the self-reported substitutions for these we examined exhibits only moderate agreement. We conclude that self-report using the measures we evaluated cannot be taken for granted to stand in for their performance-based counterparts.

Varying patterns of association observed across discordantly/concordantly frail groups were revealing. Sometimes characteristics were strongly distinguished for frailty found by SPFP criteria, whether frailty also was found by the original PFP or not. This was this case, for example, with RA prevalence in women (similar for those found frail by SPFP only and found frail by both versions, each considerably higher than for those found frail by the original PFP alone). The converse also was observed: mean age was similar for those found frail by the original PFP alone and those found frail by both versions, with each considerably higher than for those found frail by SPFP alone. In these cases, one or the other mode of assessment appears susceptible to influences beyond frailty. In other cases, a stepwise relationship was observed. This was the case, for example, for depression, where the mean symptomatology increased across those frail by the original PFP only, frail by SPFP only, and frail by both methods. In these cases, where prevalence of a characteristic seems to accumulate when frailty is identified by both methods, different ramifications of such a characteristic may be reflected in the distinct modes of assessment.

To interpret our observed between-version agreement simply as “moderate” may be criticized as over-simplifying. Kappa values of nearly 0.60 approach a range 0.60-0.0.80 commonly labeled as “substantial” [30]: Thus our suggestion of inadequate performance may be seen by some as overly pessimistic. On the other hand, standard kappa cutoffs often leave a great deal of gap in interpretation. The Kappa statistic is sensitive to the prevalence of the condition under study, hence to the extent of agreement by chance [27]. If such agreement is negligible, then Kappa is approximately the overall agreement—for which a level of 60% arguably is not impressive. Kappa indeed has been criticized for designating agreement as “substantial” in some scenarios where the overall agreement is low [31]. If, on the other hand, chance agreement is substantial, then observed agreement achieving 60% of the possible remainder may be impressive. For our overall frailty phenotype comparison between versions, the by-chance overall (unweighted) agreement was 42%; it was 71% for the observed data—an extent of improvement that is numerically “moderate”. The second point is that Kappa in the present context is meant to judge not only agreement in its own right, but accuracy with which a proxy measure replicates a gold standard. Misclassification of 29% represents a considerable measurement error, standing to introduce considerable bias in estimating relationships with potential determinants or outcomes. In summary, we consider “moderate” as a reasonable rating of agreement achieved in the present case. We also would reiterate the study’s primary finding that populations deemed as frail by the two versions differ systematically, which arguably is more concerning than a failure of agreement that could be seen as simple measurement error.

Studies on discrepancies between self-reported and performance-based measures of functioning in different settings have found considerable disagreement in classifying functional status [32–36], which speaks to the complexity of late life functioning. We hypothesize that the original PFP and the fully self-reported PFPs we examined identify systematically different “frail” populations because objective and self-reported measures of slowness and weakness target different concepts and constructs—one, physical and the other, partly psychosocial [37]. Multiple studies have evidenced strong influences of psychosocial factors on self-report [32, 38–40]. The employment of coping strategies, moreover, may either mitigate or exacerbate the impact of functional limitation depending on the social and physical context in which activities of living actually take place [41, 42]. As such, the substitution of self-reported measures for weakness and slowness in the PFP could have unintended consequences of expanding the scope of frailty assessment beyond the physical domain to also include social and psychological vulnerabilities. This expansive view of frailty and its measurement remains debatable and is beyond the scope of this paper. Beyond conceptual differences, it is also important to consider differences in the constructs targeted by our self-reported replacement items versus their objective counterparts—difficulty in tasks versus performance in task components. At the very least, successful self-reported “replacements” for objective PFP criteria likely will require deliberate design to target equivalent constructs as the objective criteria do, and not merely employ readily available task difficulty items. Research teams in Europe recently have made advancements—seeking to approximate PFP criteria employing multiple self-report items rather than one, much as we attempted here, [43] or querying changes in physical performance over time [44]. Others in our research group recently assessed agreement when making PFP item substitutions grounded in both current function and changes in function [45]. Further such work should have high priority given that there is a clear need for self-reported assessments of frailty.

We are concerned by the male–female discrepancy in the prevalence of slowness and weakness for self-reported versus performance-based assessment, because this suggests considerably differential sensitivity by sex of the original PFP versus those employing standard self-reported disability items for ascertaining frailty. Various mechanisms for the discrepancy are possible. First, self-report of functional difficulty in older adults may be subject to differential item functioning (DIF) by gender—as might occur, for example, if men were less “willing” to report difficulty than women or there was role sensitivity by sex. There are reports of such [46, 47]—but DIF has been most evident in Instrumental Activities of Daily Living (IADL) and isolated Activities of Daily Living items, and not been appreciable in mobility tasks [48]. Secondly, if the concepts and constructs assessed by self-reported versus objective criteria differ, there may be true male–female differences in the concepts and constructs measured by one mode relative to the other. Finally, men identified by the original PFP cutoffs for slowness and weakness may actually be more highly functional than women identified by these cutoffs. The cutoffs are sex-specific: This is reasonable given sex differences in height, limb length and muscle mass—but it is not clear that a 20^th^ percentile cutoff in each group identifies clinically comparable functional levels. Whatever the source of the discrepancy, work to better understand modal differences in measurement are needed if a valid self-reported PFP is to be developed, and possibly to refine the validity of the original PFP.

In defining self-reported criteria for slowness and weakness, we prioritized items having a reasonable prevalence match to the objective PFP criteria. To do otherwise “builds in” a failure of agreement and, likely, a systematic difference in functional level of populations identified. Our choice can well be debated, however, as can the original PFP definition of slowness and weakness criteria by a percentile (lowest 20^th^) rather than a clinical standard of impaired performance. There is reason to consider 20^th^ percentile cutoffs as reasonable—for slowness defined by usual gait speed, for example, these were between 0.65 m/sec (for shorter individuals) and 0.76 m/sec (for taller individuals) in our sample and paralleled commonly used cutoffs between 0.6 and 0.8 m/sec [49]. Yet, particularly having in mind sex differences discussed just above, a clinical rather than a percentile-based benchmark merits consideration. Such a benchmark might, for example, seek to optimize sensitivity and specificity for predicting a relevant adverse event such as incident dependence or need for use of an assistive device in the coming year. Other criteria such as disability items’ test–retest reliability also could be considered. Relatively few studies have evaluated this: Those which have indicate moderate-to-high reliability of the items we employed. For example, a reliability substudy of the Women’s Health and Aging study in which test–retest was evaluated several hours apart found Kappa values of 1 for difficulty walking half a mile (95% CI 0.78 to 1.00; *n* = 64) and of 0.85 for gripping with fingers (95% CI 0.61 to 1.00; *n* = 69); each, comparing any difficulty vs no difficulty [50]. In that same cohort, a variety of self-reported measures exhibited very high odds ratios for short-term agreement in a substudy in which participants were evaluated weekly for 26 weeks [51]. The National Health and Aging Trends study also conducted a reliability substudy in which *n* = 111 participants were re-interviewed 2–4 weeks apart; test–retest Kappa for walking 3 blocks was 0.75 (vs. 0.64 for climbing 10 stairs); this was 0.53 for gripping with fingers and 0.59 for lifting and carrying 10 pounds (vs. 0.57 for reaching overhead) [52]. Existing reports, then, have not identified common self-reported disability items of relevance exhibiting considerably superior reliability to those we selected. There are various assessments of frailty outside the PFP paradigm that require no performance-based assessment (e.g., the self-reported FRAIL scale [53] and Vulnerable Elders Survey [54], the clinician-assessed Clinical Frailty Scale [55], measures grounded in electronic health records [56] or claims data [57]). One might then ask: Why not simply use one of these? For some purposes, this may suffice. It has been well evidenced, however: Measures based on different paradigms identify different individuals as frail to a large degree [58–60]. These also correspond to distinct concepts and theories as to the identity of frailty [61]. Thus, they are not exchangeable, and one should be chosen based on the construct it is intended to measure and the purpose it is meant to serve [13]. The physiological specificity underlying the phenotype offers benefits if the goal is to elucidate mechanisms and etiology [62], hence we believe it will offer the best choice in some situations. Then, development of an assessment more broadly applicable in clinical settings becomes a worthy goal.

Our study’s strengths include that it was conducted using data from a large, outstandingly characterized epidemiological cohort and the same in which the PFP was developed and first validated. The missing data percentage was extremely low. We evaluated construct validity, moreover, and not only criterion validity. The latter is the type that has been most often assessed for frailty measures—as either concurrent validity (agreement such as Kappa statistics quantitate) or predictive validity (associations with frailty-related outcomes). To determine whether two assessment methods identify the same population addresses a hypothesis that must be true if the methods measure the same entity, hence assesses the former.

A primary weakness of our study is the limited number of self-reported proxies for slowness and weakness that were available for consideration. Our study, however, did not seek to identify an optimal proxy, but rather to mimic what others largely have done in their prior substitutions, so as to justify (or not) the need for better alternative substitution items. Our choices were comparable to those used in most other self-reported versions of the PFP reviewed by Theou and colleagues [14]. Secondly, our data suffered from the same limitations typical in epidemiologic cohorts, in which there may be selection bias for study participation and against those who drop out of the study prematurely. The latter may be a particular concern for frailty, as those becoming frail may well opt to not engage in a lengthy in-clinic assessment protocol such as there was for the CHS. To have warped findings distinguishing subpopulations identified as frail by different measures, however, would require that non-participants “counterbalance” differences evidenced in our study (e.g., persons in poor health not participating predominantly be frail by the objective PFP only and not the self-report version). This seems unlikely to us. To have warped findings regarding agreement would require that measures considerably more strongly agree in non-participants than participants; this seems possible. Our sample size was considerable but not large by today’s standards: Power to identify differences for men was less than for women and highly multivariate characterization of differences was not possible—hence we opted for a relatively simple analytic approach. The PFP exhaustion criterion employs two questions from the selfsame instrument used to assess depression in the CHS—the CES-D-10. Thus, there is some circularity in any depression association with frailty. Both the original PFP and our SPFP incorporated this exhaustion measure, however, and so excess symptomatology among those deemed frail by the self-reported version remains compelling in our view. As a study to identify distinctions, finally, our study made multiple comparisons of variables between study groups we defined. We took care to only highlight those findings most strongly evidenced—in crude analyses, at a Bonferroni-corrected level of 0.0015 (0.05 divided by 17 measures times 2 sexes). Follow up studies to build on and replicate (or contradict) our findings would have value.

A final concern, raised by a reviewer, merits special consideration. The CHS study is growing old; data for the sample we analyzed was collected more than 30 years ago. If self-reporting behavior has changed over the years—due either to changes in underlying frailty prevalence or to changes in the relationship of frailty to reporting, one might question the relevance of our findings to the present day. Our Kappa sensitivity analysis substituting 2015 prevalence estimates for the observed CHS prevalence showed little impact of increasing prevalence of frailty on the Kappa estimates. According to the simulation study by Byrt et al. [27], the impact of assessment bias tends to diminish as the PABAK increases and become negligible when PABAK = 0.8 or higher. Therefore, it is reasonable to conclude that the impact of changing frailty prevalence and/or self-report behavior should be minor in the community setting. Lessening over time in differential self-report of disability by personal characteristics could indeed impact findings of systematic differences in populations designated frail by PFP versus SPFP. We could not find evidence in the literature to indicate that major such differences have occurred, however, rather than simple differences in the prevalence of self-reported difficulty.

In conclusion, our study cautions against considering frailty instruments substituting self-reported disability measures for slow walk and weakness as approximate replicates of the original PFP. Self-reported PFP versions may have merit, but they then should be selected and judged for their purpose of use and recognize a distinct target of measurement. Meanwhile, our study affirms the need to develop self-reported substitutions for slow walk and weakness in the PFP that more validly approximate their objectively measured counterparts.

## Supplementary Information

**Additional file 1: Supplemental Table S1.** Agreement between physical frailty phenotypes: original assessment versus with self-reported substitutions. **Supplemental Table S2.** Agreement between physical frailty phenotypes in the hypothetical sample^1^: original assessment versus with self-reported substitutions. **Supplemental Table S3.** Characteristics comparison across females judged frail by either method only or by both^#^. **Supplemental Table S4.** Characteristics comparison across males judged frail by either method only or by both^#^.

## Data Availability

External researchers and other agencies interested in project-generated data, survey instruments, or other research methodology and procedures can obtain this information through collaborative agreements with the Principal Investigator and the Data Coordinating Center for the Cardiovascular Health Study.

## Abbreviations

BMI: Body Mass Index
CES-D: Center for Epidemiological Studies-Depression
CHF: Congestive heart failure
DIF: Differential item functioning
IADL: Instrumental Activities of Daily Living
MI: Myocardial infarction
MMSE: Mini-Mental State Examination
PABAK: Prevalence and bias-adjusted kappa
PFP: Physical frailty phenotype
ROC: Receiver operating characteristic
SPFP: Self-reported physical frailty phenotype

## References

[1] Prince MJ, Wu F, Guo Y, Gutierrez Roblado LM, O’Donnell M, Sullivan R, Yusuf S (2015). The burden of disease in older people and implications for health policy and practice. Lancet.

[2] Ferrucci L, Kuchel GA (2021). Heterogeneity of aging: individual risk factors, mechanisms, patient priorities, and outcomes. J Am Geriatr Soc.

[3] Nguyen QD, Moodie EM, Forget MF, Desmarais P, Keezer MR, Wolfson C (2021). Health heterogeneity in older adults: exploration in the Canadian longitudinal study on aging. J Am Geriatr Soc.

[4] Morley JE, Vellas B, van Kan GA, Anker SD, Bauer JM, Bernabei R, Cesari M, Chumlea WC, Doehner W, Evans J, Fried LP, Guralnik JM, Katz PR, Malmstrom TK, McCarter RJ, Gutierrez Robledo LM, Rockwood K, von Haehling S, Vandewoude MF, Walston J (2013). Frailty consensus: a call to action. J Am Med Dir Assoc.

[5] Clegg A, Young J, Iliffe S, Rikker MO, Rockwood K (2013). Frailty in elderly people. Lancet.

[6] Robinson TN, Walston JD, Brummel NE (2015). Frailty for surgeons: review of a national institute on aging conference on frailty for specialists. J Am Coll Surg.

[7] McAdams-DeMarco MA, Law A, King E (2015). Frailty and mortality in kidney transplant recipients. Am J Transplant.

[8] Damluji AA, Forman DE, van Diepen S, Alexander KP, Page RL, Hummel SL, Menon V, Katz JN, Albert NM, Afilalo J, Cohen MG, American Heart Association Council on Clinical Cardiology and Council on Cardiovascular and Stroke Nursing (2020). Older Adults in the Cardiac Intensive Care Unit: Factoring Geriatric Syndromes in the Management, Prognosis, and Process of Care: A Scientific Statement From the American Heart Association. Circulation.

[9] Guida JL, Agurs-Collins T, Ahles TA, Campisi J, Dale W, Demark-Wahnefried W, Dietrich J, Fuldner R, Gallicchio L, Green PA, Hurria A, Janelsins MC, Jhappan C, Kirkland JL, Kohanski R, Longo V, Meydani S, Mohile S, Niedernhofer LJ, Nelson C, Perna F, Schadler K, Scott JM, Schrack JA, Tracy RP, van Deursen J, Ness KK (2021). Strategies to prevent or remediate cancer and treatment-related aging. J Natl Cancer Inst.

[10] Fried LP, Tangen CM, Walston J, Newman AB, Hirsch C, Gottdiener J, Seeman T, Tracy R, Kop WJ, Burke G, McBurnie MA, Cardiovascular Health Study Collaborative Research Group (2001). Frailty in older adults: evidence for a phenotype. J Gerontol A Biol Sci Med Sci.

[11] Fried LP, Cohen AA, Xue QL, Walston J, Bandeen-Roche K, Varadhan R (2021). The physical frailty syndrome as a transition from homeostatic symphony to cacophony. Nat Aging.

[12] Kim H, Higgins PA, Canaday DH, Burant CJ, Hornick TR (2014). Frailty assessment in the geriatric outpatient clinic. Geriatr Gerontol Int.

[13] Buta BJ, Walston JD, Godino JG, Park M, Kalyani RR, Xue QL, Bandeen-Roche K, Varadhan R (2016). Frailty assessment instruments: systematic characterization of the uses and contexts of highly-cited instruments. Ageing Res Rev.

[14] Theou O, Cann L, Blodgett J, Wallace LM, Brothers TD, Rockwood K (2015). Modifications to the frailty phenotype criteria: systematic review of the current literature and investigation of 262 frailty phenotypes in the survey of health, ageing, and retirement in Europe. Ageing Res Rev.

[15] Gobbens RJ, van Assen MA, Luijkx KG, Wijnen-Sponselee MT, Schols JM (2010). The Tilburg frailty indicator: psychometric properties. J Am Med Dir Assoc.

[16] Barreto Pde S, Greig C, Ferrandez AM (2012). Detecting and categorizing frailty status in older adults using a self-report screening instrument. Arch Gerontol Geriatr.

[17] Ambagtsheer RC, Thompson MQ, Archibald MM, Casey MG, Schultz TJ (2020). Diagnostic test accuracy of self-reported screening instruments in identifying frailty in community-dwelling older people: a systematic review. Geriatr Gerontol Int.

[18] Fried LP, Borhani NO, Enright P (1991). The cardiovascular health study: design and rationale. Ann Epidemiol.

[19] Tell GS, Fried LP, Hermanson B, Manolio TA, Newman AB, Borhani NO (1993). Recruitment of adults 65 years and older as participants in the cardiovascular health study. Ann Epidemiol.

[20] Guralnik JM, Simonsick EM, Ferrucci L (1994). A short physical performance battery assessing lower extremity function: association with self-reported disability and prediction of mortality and nursing home admission. J Gerontol.

[21] Bandeen-Roche K, Xue QL, Ferrucci L, Walston J, Guralnik JM, Chaves P, Zeger SL, Fried LP (2006). Phenotype of frailty: characterization in the women's health and aging studies. J Gerontol A Biol Sci Med Sci.

[22] Ives DG, Fitzpatrick AL, Bild DE, Psaty BM, Kuller LH, Crowley PM, Cruise RG, Theroux S (1995). Surveillance and ascertainment of cardiovascular events. The cardiovascular health study. Ann Epidemiol.

[23] Andresen EM, Malmgren JA, Carter WB, Patrick DL (1994). Screening for depression in well older adults: evaluation of a short form of the CES-D. Am J Prev Med.

[24] Folstein MF, Folstein SE, McHugh PR (1975). "Mini-mental state". A practical method for grading the cognitive state of patients for the clinician. J Psychiatr Res.

[25] Cai L, Choi K, Hansen M, Harrell L (2016). Item response theory. Ann Rev Stat Appl.

[26] Hastie TJ, Tibshirani RJ (1990). Generalized additive models.

[27] Byrt T, Bishop J, Carlin JB (1993). Bias, prevalence and kappa. J Clin Epidemiol.

[28] Vannest KJ, Parker RI, Gonen O, Adiguzel T (2016). Single case research: web based calculators for SCR analysis (Version 2.0) [Web-based application].

[29] Bandeen-Roche K, Seplaki CL, Huang J, Buta B, Kalyani RR, Varadhan R, Xue QL, Walston JD, Kasper JD (2015). Frailty in older adults: a nationally representative profile in the United States. J Gerontol A Biol Sci Med Sci.

[30] Landis JR, Koch GG (1977). The measurement of observer agreement for categorical data. Biometrics.

[31] McHugh ML (2012). Interrater reliability: the kappa statistic. Biochem Med (Zagreb).

[32] Bean JF, Olveczky DD, Kiely DK, LaRose SI, Jette AM (2011). Performance-based versus patient-reported physical function: what are the underlying predictors?. Phys Ther.

[33] Sager MA, Dunham NC, Schwantes A, Mecum L, Halverson K, Harlowe D (1992). Measurement of activities of daily living in hospitalized elderly: a comparison of self-report and performance-based methods. J Am Geriatr Soc.

[34] Nielsen LM, Kirkegaard H, Østergaard LG, Bovbjerg K, Breinholt K, Maribo T (2016). Comparison of self-reported and performance-based measures of functional ability in elderly patients in an emergency department: implications for selection of clinical outcome measures. BMC Geriatr.

[35] Silva AG, Queirós A, Sa-Couto P, Rocha NP (2015). Self-reported disability: association with lower extremity performance and other determinants in older adults attending primary care. Phys Ther.

[36] Torstveit AH, Løyland B, Grov EK, Guren M, Paul SM, Ritchie C, Vistad I, Miaskowski C, Utne I (2021). Distinctions between self-report and performance-based measures of physical function in older patients prior to chemotherapy. Cancer Nurs.

[37] Jette AM, Branch LG (1985). Impairment and disability in the aged. J Chronic Dis.

[38] Kelly-Hayes M, Jette AM, Wolf PA, D'Agostino RB, Odell PM (1992). Functional limitations and disability among elders in the Framingham study. Am J Public Health.

[39] Mendes de Leon CF, Seeman TE, Baker DI, Richardson ED, Tinetti ME (1996). Self-efficacy, physical decline, and change in functioning in community-living elders: a prospective study. J Gerontol Soc Sci.

[40] Becofsky K, Baruth M, Wilcox S. Arthritis. 2013;2013:525761. 10.1155/2013/525761. Epub 2013 Sep 5. 10.1155/2013/525761 PMC3777208 24093063

[41] Feinstein AR, Josephy BR, Wells CK (1986). Scientific and clinical problems in indexes of functional disability. Ann Intern Med.

[42] Glass TA (1998). Conjugating the "Tenses" of function: discordance among hypothetical, experimental, and enacted function in older adults. Gerontologist.

[43] Op Het Veld LPM, de Vet HCW, van Rossum E, Kempen G, van Kuijk SMJ, Beurskens A (2018). Substitution of Fried's performance-based physical frailty criteria with self-report questions. Arch Gerontol Geriatr.

[44] Nunes DP, Duarte YA, Santos JL, Lebrao ML (2015). Screening for frailty in older adults using a self-reported instrument. Rev Saude Publica.

[45] Buta B, Zheng S, Langdon J, Adeosun B, Bandeen-Roche K, Walston J, Xue QL (2022). Agreement between standard and self-reported assessments of physical frailty syndrome and its components in a registry of community-dwelling older adults. BMC Geriatr.

[46] Fleishman JA, Spector WD, Altman BM (2002). Impact of differential item functioning on age and gender differences in functional disability. J Gerontol B Psychol Sci Soc Sci.

[47] Wæhrens EE, Kottorp A, Nielsen KT (2021). Measuring self-reported ability to perform activities of daily living: a Rasch analysis. Health Qual Life Outcomes.

[48] Roorda LD, Green JR, Houwink A, Bagley PJ, Smith J, Molenaar IW, Geurts AC (2012). The Rivermead Mobility Index allows valid comparisons between subgroups of patients undergoing rehabilitation after stroke who differ with respect to age, sex, or side of lesion. Arch Phys Med Rehabil.

[49] Abellan van Kan G, Rolland Y, Andrieu S (2009). Gait speed at usual pace as a predictor of adverse outcomes in community-dwelling older people. J Nutr Health Aging.

[50] Fried LP, Bandeen-Roche K, Williamson JD, Prasada-Rao P, Chee E, Tepper S, Rubin GS (1996). Functional decline in older adults: expanding methods of ascertainment. J Gerontol A Biol Sci Med Sci.

[51] Rathouz PJ, Kasper JD, Zeger SL, Ferrucci L, Bandeen-Roche K, Miglioretti DL, Fried LP (1998). Short-term consistency in self-reported physical functioning among elderly women: the women's health and aging study. Am J Epidemiol.

[52] Freedman VA, Kasper JD, Cornman JC, Agree EM, Bandeen-Roche K, Mor V, Spillman BC, Wallace R, Wolf DA (2011). Validation of new measures of disability and functioning in the national health and aging trends study. J Gerontol A Biol Sci Med Sci.

[53] Morley JE, Malmstrom TK, Miller DK (2012). A simple frailty questionnaire (FRAIL) predicts outcomes in middle aged African Americans. J Nutr Health Aging.

[54] Saliba D, Elliott M, Rubenstein LZ, Solomon DH, Young RT, Kamberg CJ, Roth C, MacLean CH, Shekelle PG, Sloss EM, Wenger NS (2001). The vulnerable elders survey: a tool for identifying vulnerable older people in the community. J Am Geriatr Soc.

[55] Rockwood K, Song X, MacKnight C, Bergman H, Hogan DB, McDowell I, Mitnitski A (2005). A global clinical measure of fitness and frailty in elderly people. Canad Med Assoc J.

[56] Clegg A, Bates C, Young J, Ryan R, Nichols L, Ann Teale E, Mohammed MA, Parry J, Marshall T (2016). Development and validation of an electronic frailty index using routine primary care electronic health record data. Age Ageing.

[57] Kim DH, Schneeweiss S, Glynn RJ, Lipsitz LA, Rockwood K, Avorn J (2018). Measuring frailty in medicare data: development and validation of a claims-based frailty index. J Gerontol A Biol Sci Med Sci.

[58] Cigolle CT, Ofstedal MB, Tian Z, Blaum CS (2009). Comparing models of frailty: the health and retirement study. J Am Geriatr Soc.

[59] Aguayo GA, Donneau AF, Vaillant MT (2017). Agreement between 35 published frailty scores in the general population. Am J Epidemiol.

[60] Xue QL, Tian J, Walston JD, Chaves PHM, Newman AB, Bandeen-Roche K (2020). Discrepancy in frailty identification: move beyond predictive validity. J Gerontol A Biol Sci Med Sci.

[61] Bandeen-Roche K, Gross AL, Varadhan R, Buta B, Carlson MC, Huisingh-Scheetz M, Mcadams-Demarco M, Piggott DA, Brown TT, Hasan RK, Kalyani RR, Seplaki CL, Walston JD, Xue QL (2020). Principles and issues for physical frailty measurement and its clinical application. J Gerontol A Biol Sci Med Sci.

[62] Walston JD, Bandeen-Roche K (2015). Frailty: a tale of two concepts. BMC Med.

