# Supplementary material for: Substitution of self-reported measures for objectively assessed grip strength and slow walk in the Physical Frailty Phenotype: ramifications for validity
Source: BMC Geriatr. 2023 Jul 22;23:451. doi: 10.1186/s12877-023-04105-8 (PMC10362666; doi:10.1186/s12877-023-04105-8)
Supplement: Supplementary file 1 — Additional file 1: Supplemental Table S1. Agreement between physical frailty phenotypes: original assessment versus with self-reported substitutions. Supplemental Table S2. Agreement between physical frailty phenotypes in the hypothetical sample1: original assessment versus with self-reported substitutions. Supplemental Table S3. Characteristics comparison across females judged frail by either method only or by both#. Supplemental Table S4. Characteristics comparison across males judged frail by either method only or by both#. [file 12877_2023_4105_MOESM1_ESM.docx]

**Supplemental Table S1 Agreement between physical frailty phenotypes: original assessment versus with self-reported substitutions**

|  | Frailty defined by objective measures | Frailty defined by self-report^1^ measures | | | Kappa (95% CI) |
| --- | --- | --- | --- | --- | --- |
|  |  | Nonfrail  (n=2755) | prefrail  (n=2501) | Frail  (n=628) |  |
| Overall | Nonfrail (n=2516) | 2016  (80.1)^2^, (73.2)^3^ | 500  (19.9), (20.0) | 0  (0.0), (0.0) | Kappa with Linear Weighting  0.55  (0.53-0.57)  PABAK^4^  0.66 (0.65-0.68) |
|  | Prefrail (n=2870) | 733  (25.5), (26.6) | 1811  (63.1), (72.4) | 326 (11.4),(51.9) |  |
|  | Frail  (n=498) | 6  (1.2), (0.2) | 190  (38.2), (7.6) | 302  (60.6), (48.1) |  |
|  |  |  |  |  |  |
| Female |  | Nonfrail  (n=1368) | prefrail  (n=1560) | Frail  (n=462) |  |
|  | Nonfrail (n=1443) | 1054  (73.0), (77.1) | 389  (27.0), (24.9) | 0  (0.0), (0.0) | Kappa with Linear Weighting  0.55  (0.53-0.58)  PABAK  0.65 (0.64-0.67) |
|  | Prefrail (n=1631) | 311  (19.1), (22.7) | 1076  (66.0), (69.0) | 244  (15.0), (52.8) |  |
|  | Frail  (n=316) | 3  (1.0), (0.2) | 95  (30.1), (6.1) | 218  (69.0), (47.2) |  |
|  |  |  |  |  |  |
| Male |  | Nonfrail  (n=1387) | prefrail  (n=941) | Frail  (n=166) |  |
|  | Nonfrail (n=1073) | 962  (89.7), (69.4) | 111  (10.3), (11.8) | 0  (0.0), (0.0) | Kappa with Linear Weighting  0.55  (0.52-0.58)  PABAK  0.68 (0.66-0.70) |
|  | Prefrail (n=1239) | 422  (34.1), (30.4) | 735  (59.3), (78.1) | 82  (6.6), (49.4) |  |
|  | Frail  (n=182) | 3  (1.7), (0.2) | 95  (52.2), (10.1) | 84  (46.2), (50.6) |  |

CI: confidence interval

^1^Weakness assessed by report of difficulty gripping with hands or transferring from a bed or chair or lifting a 10-pound bag of groceries; Slowness assessed by report of difficulty of walking one-half a mile

^2^ row percentage

^3^ column percentage

^4^ PABAK: Prevalence and Bias Adjusted Kappa that assigns partial credit of1/2 to misses by 1 category

**Supplemental Table S2 Agreement between physical frailty phenotypes in the hypothetical sample^1^: original assessment versus with self-reported substitutions**

|  | Frailty defined by objective measures | Frailty defined by self-report^2^ measures | | | Kappa (95% CI) |
| --- | --- | --- | --- | --- | --- |
|  |  | Nonfrail  (n=2905) | prefrail  (n=2476) | Frail  (n=503) |  |
| Overall | Nonfrail (n=2516) | 1927  (83.5)^3^, (71.8)^4^ | 380  (16.5), (15.2) | 0  (0.0), (0.0) | Kappa with Linear Weighting  0.58  (0.56-0.60)  PABAK^5^  0.66 (0.65-0.67) |
| (N=5884) | Prefrail (n=2870) | 742  (27.7), (27.7) | 1711  (63.9), (68.4) | 225  (8.4), (32.2) |  |
|  | Frail  (n=498) | 14  (1.6), (0.5) | 412  (45.8), (16.5) | 473  (52.6), (67.8) |  |
|  |  |  |  |  |  |
| Female |  | Nonfrail  (n=1493) | prefrail  (n=1534) | Frail  (n=363) |  |
| (N=3390) | Nonfrail (n=1443) | 945  (78.3), (72.4) | 262  (21.7), (16.7) | 0  (0.0), (0.0) | Kappa with Linear Weighting  0.59  (0.57-0.61)  PABAK  0.66 (0.64-0.67) |
|  | Prefrail (n=1631) | 351  (21.9), (26.9) | 1076  (67.3), (68.7) | 173  (10.8), (33.4) |  |
|  | Frail  (n=316) | 9  (1.6), (0.7) | 229  (39.2), (14.6) | 345  (59.2), (66.6) |  |
|  |  |  |  |  |  |
| Male |  | Nonfrail  (n=1412) | prefrail  (n=942) | Frail  (n=140) |  |
| (N=2494) | Nonfrail (n=1073) | 989  (90.6), (71.9) | 103  (9.4), (11.1) | 0  (0.0), (0.0) | Kappa with Linear Weighting  0.57  (0.55-0.60)  PABAK  0.67 (0.65-0.69) |
|  | Prefrail (n=1239) | 381  (35.3), (27.7) | 642  (59.5), (69.1) | 57  (5.3), (30.0) |  |
|  | Frail  (n=182) | 5  (1.7), (0.4) | 184  (57.1), (19.8) | 133  (41.2), (70.0) |  |

^1^cell counts estimated using the same grand totals as in the CHS (i.e., n=3390 for females and n=2494 for males) but actual prevalence of 15.3% being frail and 45.5% being prefrail overall, and 17.2% being frail and 47.2% being prefrail (by PFP) among females and 12.9% being frail and 43.3% being prefrail among males in the National Health and Aging Trends Study sample (Bandeen-Roche et al., J Gerontol A Biol Sci Med Sci, 2015, Vol. 70, No. 11, 1427–1434).

^2^ Weakness assessed by report of difficulty gripping with hands or transferring from a bed or chair; Slowness assessed by report of difficulty of walking one-half a mile

^3^ row percentage

^4^ column percentage

^5^ PABAK: Prevalence and Bias Adjusted Kappa that assigns partial credit of1/2 to misses by 1 category

**Supplemental Table S3. Characteristics comparison across females judged frail by either method only or by both^#^**

|  | Frail by self-report only (n=176) | Frail by objective only (n=129) | Frail by both  (n=187) | p-value* |
| --- | --- | --- | --- | --- |
| Age (years), mean (std) | 73.8 (6.4) | 76.5 (6.6) | 76.3 (6.1) | <0.01 |
| Education (years), mean (std) | 12.1 (4.5) | 12.2 (5.1) | 12.1 (4.8) | 0.98 |
| MMSE, mean (std) | 88.2 (7.5) | 82.6 (13.0) | 85.6 (8.6) | <0.01 |
| # of disease*, mean (std) | 2.1 (1.5) | 1.5 (1.2) | 2.1 (1.4) | <0.01 |
| BMI, mean (std) | 30.2 (6.7) | 26.5 (6.1) | 27.3 (7.0) | <0.01 |
| Race(black), n (%) | 33 (18.8) | 47 (36.4) | 52 (27.8) | <0.01 |
| Marriage, n (%)  Married  Widowed  Separated/divorced/never married | 100 (56.8)  58 (33.0)  18 (10.2) | 45 (34.9)  58 (45.0)  26 (20.2) | 73 (39.0)  86 (46.0)  28 (15.0) | <0.01 |
| # of depressive symptoms, mean (std) | 9.2 (5.6) | 6.7 (5.3) | 10.9 (5.9) | <0.01 |
| Income  <$16000  $16000-35000  >$35000 | 99 (55.3)  47 (28.1)  21 (12.6) | 87 (73.1)  16 (13.5)  16 (13.5) | 115 (68.1)  37 (21.9)  17 (10.1) | 0.05 |
| Health status, n (%)  excellent  very good  good  fair  poor | 5 (2.8)  14 (8.0)  46 (26.1)  82 (46.6)  29 (16.5) | 5 (3.9)  17 (13.3)  42 (32.8)  55 (43.0)  9 (7.0) | 4 (2.1)  16 (8.6)  35 (18.7)  79 (42.3)  53 (28.3) | <0.01 |
| Cancer, n (%) | 35 (19.9) | 13 (10.1) | 35 (18.7) | 0.05 |
| Congestive heart failure, n (%) | 19 (10.8) | 12 (9.3) | 28 (15.0) | 0.26 |
| Myocardial infarction, n (%) | 21 (11.9) | 12 (9.3) | 17 (9.1) | 0.62 |
| Coronary heart disease, n (%) | 50 (28.4) | 26 (20.2) | 58 (31.0) | 0.09 |
| Rheumatoid arthritis, n (%) | 144 (82.3) | 82 (64.6) | 152 (84.0) | <0.01 |
| Diabetics, n (%) | 60 (34.5) | 39 (31.0) | 70 (38.9) | 0.35 |
| Angina, n (%) | 45 (25.6) | 21 (16.3) | 52 (27.8) | 0.05 |

# Weakness self-reported substitution = transferring, gripping, and lifting.

* For continuous variables, ANOVA was used to calculate and for categorical variables, Chi-square test was used.

**Supplemental Table S4. Characteristics comparison across females judged frail by either method only or by both^#^**

|  | Frail by self-report only (N=65) | Frail by objective only (N=107) | Frail by both  (N=75) | p-value* |
| --- | --- | --- | --- | --- |
| Age (years), mean (std) | 74.1 (6.0) | 77.5 (6.6) | 77.2 (6.7) | <0.01 |
| Education (years), mean (std) | 12.0 (5.1) | 12.9 (5.1) | 12.1 (5.1) | 0.42 |
| MMSE, mean (std) | 85.7 (7.6) | 84.9 (9.9) | 84.2 (11.9) | 0.70 |
| # of disease*, mean (std) | 2.3 (1.5) | 1.9 (1.4) | 2.5 (1.7) | 0.02 |
| BMI, mean (std) | 26.5 (4.1) | 26.2 (4.0) | 26.1 (4.5) | 0.78 |
| Race(black), n (%) | 11 (16.9) | 29 (27.0) | 12 (16.0) | 0.12 |
| Marriage, n (%)  Married  Widowed  Separated/divorced/never married | 50 (76.9)  10 (15.4)  5 (7.7) | 79 (73.8)  17 (15.9)  11 (10.3) | 51 (68.0)  17 (22.7)  7 (9.3) | 0.71 |
| # of depressive symptoms, mean (std) | 7.1 (5.1) | 6.6 (4.7) | 9.1 (6.0) | <0.01 |
| Income  <$16000  $16000-35000  >$35000 | 34 (54.8)  22 (35.5)  6 (9.7) | 46 (46.0)  33 (33.0)  21 (21.0) | 33 (45.2)  27 (37.0)  13 (17.8) | 0.42 |
| Health status, n (%)  excellent  very good  good  fair  poor | 1 (1.6)  2 (3.2)  23 (36.5)  20 (31.7)  17 (27.0) | 3 (2.8)  12 (11.2)  48 (44.9)  39 (36.4)  5 (4.7) | 0 (0)  8 (10.7)  19 (25.3)  26 (34.7)  22 (29.3) | <0.03 |
| Cancer, n (%) | 12 (18.5) | 17 (15.9) | 10 (13.5) | 0.73 |
| Congestive heart failure, n (%) | 12 (18.5) | 9 (8.4) | 17 (22.7) | 0.02 |
| Myocardial infarction, n (%) | 15 (23.1) | 17 (15.9) | 20 (26.7) | 0.19 |
| Coronary heart disease, n (%) | 22 (33.8) | 26 (24.3) | 33 (44.0) | 0.02 |
| Rheumatoid arthritis, n (%) | 49 (75.4) | 58 (55.2) | 54 (73.0) | <0.01 |
| Diabetics, n (%) | 21 (32.1) | 50 (47.6) | 29 (39.7) | 0.14 |
| Angina, n (%) | 17 (26.2) | 22 (20.6) | 28 (37.3) | 0.04 |

# Weakness self-reported substitution = transferring, gripping, and lifting.

* For continuous variables, ANOVA was used to calculate and for categorical variables, Chi-square test was used.
